# Diversity of Apolipoprotein E genetic polymorphism significance on cardiovascular risk is determined by the presence of Metabolic Syndrome among hypertensive patients

**DOI:** 10.1186/1476-511X-13-174

**Published:** 2014-11-20

**Authors:** Andrei Alkmim Teixeira, Mauro Sergio Marrocos, Beata Marie Redublo Quinto, Maria Aparecida Dalboni, Cassio Jose de Oliveira Rodrigues, Silmara de Melo Carmona, Mariana Kuniyoshi, Marcelo Costa Batista

**Affiliations:** Nephrology Division, R. Pedro de Toledo, Universidade Federal de São Paulo, 781 14o. andar, Vila Clementino, São Paulo, São Paulo CEP 04039-032 Brazil; Nephrology Division-New England Medical Center, Tufts University, Boston, Massachusetts USA; Research and Education Institute, Hospital Israelita Albert Einstein, São Paulo, São Paulo Brazil

**Keywords:** Apolipoprotein E, Polymorphism, Cardiovascular risk, Metabolic syndrome, Hypertension

## Abstract

**Background:**

Hypertension has a significant relevance as a cardiovascular risk factor. A consistent increase on world’s Metabolic Syndrome (MetS) incidence has been associated with an epidemic cardiovascular risk in different populations. Dislipidemia plays a major role determining the epidemic CV burden attributed to MetS. Apolipoprotein E (ApoE) is involved on cholesterol and triglycerides metabolism regulation. Once ApoE polymorphism may influence lipid metabolism, it is possible that it brings on individual susceptibility consequences for the development of MetS and cardiovascular risk. The objective of the study is to measure the discriminatory power of ApoE polymorphism in determining cardiovascular risk stratification based on the presence MetS in a cohort of hypertensive patients.

**Methods:**

It was enrolled 383 patients, divided in two groups, classified by MetS presence (IDF criteria): Group 1: 266 patients with MetS (MetS +) and Group 2: 117 patients without Mets (MetS -). Patient’s data were collected by clinical evaluation, physical exam, file reviews and laboratory testing. Polymorphic ApoE analysis was performed by PCR amplification. Groups were compared on clinical and laboratory characteristics as well as allele and genotype distribution towards ApoE polymorphism. Mets CVD prevalence was analysed according to E4 allele prevalence.

**Results:**

The results evidenced 184 men (48%), 63,7% whites, 45,1% diabetics and 11,7% of patients were smokers. Mean age was 64,0 ± 12,0 years. When genotypic distribution was analyzed, E3/3 genotype and E3 allele frequencies were more prevalent. Among patients with MetS, we observed an independent association between CVD prevalence and E4 allele frequency (OR 2.42 (1.17- 5.0, p < 0,05)). On the opposite direction, in those without MetS, there was lesser CVD burden in E4 allele carriers (OR 0,14 (0,02-0,75)). These associations remained significant even after confounding factor corrections.

**Conclusions:**

The results presented demonstrate that the association between ApoE gene and CVD may be modulated by the presence of MetS, with an increased CV burden observed among E4 allele carriers with the syndrome. On the opposite way, E4 allele carriers without visceral obesity had lesser prevalence of CVD.

## Background

Hypertension has a worldwide prevalence estimated in 1 billion of people, an attributable mortality of 7,1 million of deaths per year and a significant relevance as a cardiovascular risk factor. Intrinsically related with hypertension, visceral obesity, the main component of Metabolic Syndrome (MetS), significantly contribute to the reduction of life expectancy. According to the World Health Organization (WHO), 1,5 billion of people will be obese in 2015 [1]. Regarded as a clinicopathological clustering of metabolic disorders determining increased cardiovascular disease (CVD) risk, MetS has a polygenic inherence in which abdominal fat accumulation plays pivotal role for such epidemic morbidity [2]. Although a significant portion of CVD in MetS may be explained by the presence of well-known risk factors, a significant proportion remains unexplained possibly due to individual genetic variability.

Among those individuals with MetS, dislipidemia and its consequences (coronary artery disease, stroke and others) are attributed to a conjunct and integrative action between genetic and environmental factors. The main lipoprotein abnormality related with MetS is low concentration of HDL cholesterol and hypertriglyceridemia. The characteristic components of the syndrome are mostly determined by apolipoprotein E (ApoE) modulation of atherogenic plasma Apo-B containing lipoproteins. In fact, ApoE is the main constituent of tg-rich lipoprotein and genetic polymorphic variations of this protein has been associated with CVD occurrence. Identification of genetic markers has clinical relevance in this context, as for risk prediction as for adoption of therapeutic maneuvers.

Apo E has three different alleles (E2, E3 and E4) determining different and complex associations with clinical phenotype expressions. In fact, epidemiological studies suggested that E4 allele carriers were particularly predisposed to develop higher cholesterol levels and higher coronary artery disease (CAD) mortality, meanwhile others studies concluded E3 and E4 alellles presences were associated with higher risk in men. Conversely, *Olivieri et al.*[3] found a higher MetS prevalence among E4 alelle carriers while *Ferreira et al.*[4] did not observe such association of this allele with MetS or hypertension.

Due to the its polymorphic characteristic determining diversity in clinical expression, we speculate whether Apo E genotype could influence diversely on CVD burden based on the presence of MetS on specific high risk population. With this regard, the aim of this study is to measure the discriminatory power of ApoE genetic polymorphism in determining the cardiovascular risk stratification based on the presence MetS in a cohort of hypertensive patients.

## Results

At the end of the study, 383 patients were analyzed. Population clinical and laboratory characteristics are shown on Table 1. As depicted on Table 1, 184 patients were men (48%), 63.7% whites, 45.1% diabetics,11.7% of patients were smokers and mean age was 64,0 ± 12,0 years. Patients were classified by MetS diagnosis. MetS (+) group showed higher prevalence of men and, as expected, weight, BMI, waist, CRP, dislipidemia (including lower HDL-cholesterol and triglycerides levels) and CVD prevalence were higher in this group when comparing with Mets (−) group.

**Table 1:** **Population clinical and laboratory characteristics based on MetS presence**

	Total	MetS (+)	MetS (−)	p
		(n = 266)	(n = 117)	
Men	184 (48.0%)	117 (43.9%)	67 (57.2%)	< 0.05
Age (years)	64.0 ± 12.0	64.0 ± 11.7	63.7 ± 12.7	NS
White	244 (63.7%)	173 (65%)	71 (60.6%)	NS
Weight (kg)	78.0 ± 44.4	82.1 ± 52.2	69.0 ± 13.7	< 0.05
BMI (kg/cm2)	29.3 ± 5.6	30.6 ± 5.2	26.5 ± 5.6	< 0.05
Waist (cm)	100.2 ± 13.8	104.5 ± 11.8	90.8 ± 13.1	< 0.05
DM	173 (45.1%)	144 (54.1%)	29 (24.7%)	< 0.05
Dislipidemia	311 (81.2%)	229 (86%)	82 (70%)	< 0.05
CVD	133 (34.7%)	101 (37.9%)	32 (27.3%)	< 0.05
Sedentarism	302 (78.8%)	210 (78.9%)	92 (78.6%)	NS
Smoking	45 (11.7%)	34 (12.7%)	11 (9.4%)	NS
Alcohol	28 (7.3%)	18 (6.7%)	10 (8.5%)	NS
Chol (mg/dl)	181.5 ± 40.0	181.5 ± 41.8	181.9 ± 36.2	NS
HDL-c (mg/dl)	46.6 ± 13.0	42.6 ± 9.9	55.7 ± 14.9	< 0.05
LDL-c (mg/dl)	105.0 ± 33.5	105.0 ± 35.1	105.4 ± 30.0	NS
TG (mg/dl)	151.2 ± 83.8	170.3 ± 83.7	109.7 ± 68.7	< 0.05
Cr (mg/dl)	1.46 ± 0,86	1.46 ± 0.89	1.44 ± 0,83	NS
U (mg/dl)	58.7 ± 31.3	58.9 ± 31.7	58.5 ± 31,0	NS
CRP (mg/dl)	0.56 ± 0.77	0.64 ± 0.86	0.39 ± 0.51	< 0.05

BMI: body max index; DM: diabetes mellitus; Chol: total cholesterol; HDL-C: HDL cholesterol; LDL-c: LDL cholesterol; TG: triglycerides; Cr: creatinine; U: urea; CRP: C reactive protein. p: <0,05 MetS (+) versus Mets (−); p: NS, non significant.

As shown on Table 2, no statistical difference was identified between groups when genotypes and alleles distribution, stratified by race, were analyzed.

**Table 2:** **ApoE genotypes and alleles distribution based on race**

	White (N = 237)	Black (N = 68)	Others (N = 78)	p
E4/4	4 (1.7%)	2 (2.9%)	1 (1.3%)	NS
E4/3	41 (17.2%)	7 (10.1%)	8 (10.3%)	NS
E3/3	167 (71%)	51 (75.4%)	60 (76.8%)	NS
E3/2	20 (8.0%)	6 (8.7%)	8 (10.3%)	NS
E4/2	5 (2.1%)	2 (2.9%)	1 (1.3%)	NS
E2 allele	24 (10.1%)	8 (11.6%)	9 (11.5%)	NS
E3 allele	227 (96.2%)	63 (94.2%)	78 (97.4%)	NS
E4 allele	50 (21%)	11 (15.9%)	10 (12.8%)	NS

Genotypes: E4/4, E 4/3, E 3/3, E 3/2, E 4/2. Alleles: 2,3 and 4. p: NS, non significant.

In genotypic distribution analysis, it was identified that E3/3 genotype and E3 allele frequencies were more prevalent. E4/3 genotype and E4 allele were the second most frequent genotype and allele, respectively. This prevalence, even not statistically significant, remained similar independently of MetS presence (Table 3).

**Table 3:** **ApoE genotypes and alleles distribution based on MetS presence**

	Total	MetS (+)	Mets (−)	p
		(n = 266)	(n = 117)	
E4/4	7 (1.8%)	5 (1.9%)	2 (1.7%)	NS
E4/3	56 (14.6%)	37 (13.9%)	19 (16.2%)	NS
E3/3	278 (72.6%)	197 (73.3%)	85 (70.9%)	NS
E3/2	34 (8.9%)	24 (9.0%)	10 (8.5%)	NS
E4/2	8 (2.1%)	5 (1.9%)	3 (2.6%)	NS
E2 allele	42 (11.0%)	29 (10.9%)	13 (11.1%)	NS
E3 allele	368 (96.1%)	256 (96.2%)	112 (95.7%)	NS
E4 allele	71 (18.5%)	47 (17.7%)	24 (20.5%)	NS

Genotypes: E4/4, E 4/3, E 3/3, E 3/2, E 4/2. Alleles: 2,3 and 4. p: NS, non significant.

Analyses of E2 and E3 allleles clinical and laboratory characteristics showed no statistical difference between allele (+) versus allele (−) groups (data not shown). However, stratified by MetS presence, based on E4 allele frequency, clinical laboratory results evidenced, in MetS (+) patients, higher CVD prevalence in allele E4 carriers, meanwhile, in patients without MetS diagnosis, allele E4 presence was associated with lesser CVD prevalence (Table 4).

**Table 4:** **Population clinical and laboratory characteristics according to Mets diagnosis based on ApoE E4 allele presence**

	MetS (+) n = 266			MetS (−) n = 117		
	E4 allele (−)	E4 allele (+)	N	E4 allele (−)	E4 allele (+)	N
Men	92 (42.0%)	21 (44.7%)	NS	49 (52.7%)	14 (58.3%)	NS
Age (years)	64.4 ± 11.3	62.5 ± 12.5	NS	63.1 ± 12.7	64.8 ± 13.1	NS
White	131 (59.8%)	37 (78.7%)	< 0,05	55 (59.8%)	13 (54.2%)	NS
Weight (kg)	82.8 ± 57.7	79.0 ± 16.8	NS	68.9 ± 14.0	69.5 ± 14.0	NS
BMI (kg/cm2)	30.6 ± 4.9	30.5 ± 6.4	NS	26.6 ± 5.8	26.6 ± 5.3	NS
Waist (cm)	104.4 ± 11.6	104.6 ± 13.5	NS	91.1 ± 13.4	91.0 ± 13.0	NS
DM	114 (52.1%)	25 (53.2%)	NS	21 (22.8%)	6 (25.0%)	NS
Dislipidemia	184 (84.0%)	41 (87.2%)	NS	63 (67.7%)	16 (66.7%)	NS
CVD	75 (34.7%)	24 (51.1%)	< 0,05	27 (30.0%)	2 (8.3%)	< 0.05
Sedentarism	171 (80.7%)	34 (75.6%)	NS	69 (76.7%)	20 (87.0%)	NS
Smoking	30 (14.0%)	4 (8,5%)	NS	8 (8.7%)	3 (13.6%)	NS
Alcohol	16 (7.5%)	2 (4,3%)	NS	7 (7.7%)	3 (13.0%)	NS
Chol (mg/dl)	182.2 ± 42.2	179.4 ± 41.5	NS	186.5 ± 37.1	170.0 ± 27.5	< 0.05
HDL-c (mg/dl)	43.0 ± 9.8	40.5 ± 10.4	NS	56.2 ± 15.7	52.9 ± 11.4	NS
LDL-c (mg/dl)	105.9 ± 35.4	104.1 ± 33.2	NS	109.6 ± 31.3	93.3 ± 20.5	< 0.05
TG (mg/dl)	168.2 ± 80.1	184.3 ± 94.3	NS	108.5 ± 69.8	117.8 ± 71.6	NS
Cr (mg/dl)	1.48 ± 0.92	1.36 ± 0.70	NS	1.43 ± 0.77	1.51 ± 1.09	NS
U (mg/dl)	59.6 ± 31.9	55.4 ± 31.6	NS	57.9 ± 29.7	60.2 ± 36.2	NS
CRP (mg/dl)	0.63 ± 0.80	0.61 ± 0.81	NS	0.41 ± 0.52	0.32 ± 0.49	NS

BMI: body mass index; DM: diabetes mellitus; Chol: total cholesterol; HDL-C: HDL cholesterol; LDL-c: LDL cholesterol; TG: Triglycerides; Cr: creatinine; U: urea; CRP: C reactive protein C. p: NS, non significant.

These associations remained statistically significant even after confounding agents adjustment like age, sex, smoking, DM, total cholesterol, HDL-cholesterol and LDL-cholesterol (Table 5).

**Table 5:** **Logistic regression of CVD and allele 4 association in MetS (+) and MetS (−) patients adjusted for age, sex, smoking, DM, Col, HDL-C and LDL-C**

Metabolic syndrome (+)			
CVD	OR	CI	P
Adjusted for E4 allele	1.96	1.03 - 3.70	0.03
Adjusted for E4 allele, age	2.28	1.16 – 4.47	0.01
Adjusted forE4 allele, age, sex	2.28	1.16 – 4.49	0.01
Adjusted for E4 allele, age, sex, smoking	2.45	1.23 – 4.87	0.01
Adjusted for E4 allele, age, sex, smoking, DM	2.45	1.22 – 4.90	0.01
Adjusted for E4 allele, age, sex, smoking, DM, Col	2.37	1.16 - 4.81	0.01
Adjusted for E4 allele, age, sex, smoking, DM, Col, HDL-C	2.38	1.16 - 4.87	0.01
Adjusted for E4 allele, age, sex, smoking, DM, Col, HDL-C, LDL-C	2.23	1.08 - 4.59	0.02
**Metabolic syndrome (−)**			
**CVD**	**OR**	**CI**	**P**
Adjusted for E4 allele	0.21	0.04 – 0.96	0.04
Adjusted for E4 allele, age	0.19	0.04 – 0.90	0.03
Adjusted for E4 allele, age, sex	0.18	0.04 – 0.87	0.03
Adjusted for E4 allele, age, sex, smoking	0.20	0.04 – 0.97	0.04
Adjusted for E4 allele, age, sex, smoking, DM	0.19	0.03 - 0.92	0.04
Adjusted for E4 allele, age, sex, smoking, DM, Chol	0.17	0,03 - 0,83	0.02
Adjusted for E4 allele, age, sex, smoking, DM, Chol, HDL-C	0.17	0.03 - 0.84	0.03
Adjusted for E4 allele, age, sex, smoking, DM, Chol, HDL-C, LDL-C	0.19	0.03 - 1.00	0.05

CVD: cardiovascular disease; DM: diabetes mellitus; Chol: total cholesterol, HDL-C: HDL-cholesterol; LDL-C: LDL-cholesterol.

## Discussion

Our study of ApoE genetic polymorphism on cardiovascular stratification in a cohort of hypertensive Mets (+) patients evidenced higher CVD prevalence among those E4 allele carriers patients. However, CVD prevalence in Mets (−) E4 allele carriers was lower. This association can be influenced by the presence of traditional cardiovascular risk factors [5].

In *Chu et al.*[6], E2 allele was described as the rarest allele in several races, and E4 allele was described as 15% in white and 22% in non hispanic black. The most common genotypes E2/3, E3/3 and E3/4 amounted 95% of white genotypes and 89% of blacks. In our study, similarly to the literature, E2 allele was less prevalent and E4 allele was present 21% of whites and 15,9% of black patients. E3/2, E3/3 and E4/3 genotypes amounted 96,2% in white and 94,2% in black. When we analyzed the alleles and genotypes distribution stratified by race, we did not find statistical differences between groups.

In comparisons looking for risk determination, E3/3 homozygote genotype has been used as reference. In general, E2 allele presence is associated to lower cholesterol and LDL-cholesterol levels [7] and triglycerides elevation [8]. On the other hand, E4 allele is associated with total cholesterol and LDL-cholesterol elevation. These effects were demonstrated in several populations, including pediatrics ones [9]. However, we did not find clinical and laboratory differences in patients stratified by alleles 2, 3 and 4 presence (data not shown). The genotypic and allelic distribuition were similar to the observed in other publications [10]. E3/3 genotype was the most prevalent genotype globally and between Mets (+) and MetS (−) groups, as well as when E3 allele was analysed.

Epidemiological studies analysed ApoE polymorphism impact on cardiovascular disease. *Eichner et al.*[11] and *Lehtinen et al.*[12] suggested that E4 allele carriers were particularly predisposed to develop coronary lesions or to possess elevated risk of CAD death. The authors suggest that this could be a consequence of lipoprotein metabolism dysfunction associated to E4 isoform, with elevation of total cholesterol and triglycerides levels [13]. More recently, similar results was described by *Chaudhary et al.*[14], in a type 2 diabetes mellitus population. *Schiele et al.*[15], studying northern europe populations with higher cholesterol levels and higher CVD mortality have shown higher E4 alelle presence also. *Hixson et al.*[16] and *Ilveskoski et al.*[17], studying vascular necropsy alterations, showed more atherosclerotic lesions in E4 allele carriers, independently of total cholesterol levels. On the other hand, angiographic studies did not evidenced higher CAD risk in E4 allele carriers [18].

In *MONICA study*[19], E4 allele frequency elevation of 0,01 was associated to a rise on CAD death of 24,5 per 100.000 patients. In 4S study [20], E4 allele carriers presented 80% higher death risk after myocardial infarction, in comparison with other patients. *Lahoz et al.*[21] concluded that E2 and E4 allele presences were associated with higher cardiovascular risk in men. The authors argued that E4 allele determine higher risk partly due to the lipid alterations that it brings on.

In our study, although E4 allele had not been associated with higher MetS prevalence neither conferred higher cardiovascular disease risk on study population, when analyzing exclusively MetS population, we identified an association between CVD and E4 allele. On the other hand, when we analysed Mets (−) population, we identified higher prevalence of E4 allele in CVD negative than CVD positive patients. These associations remained significant even after correction for confounding factors. This result seem to suggest that E4 allele capability to determine higher prevalence of CVD would be entailed by MetS presence, corroborating Apolipoprotein E modulation of atherogenic plasma Apo-B containing lipoproteins seen in this disorder. Some studies have described a controversial role of E4 allele polymorphism in different populations, some depositing a protector role and others an adverse outcome related with its presence. *Yan et al.*[22] described a protector polymorphism effect on the development of foam liver disease; ARIC study researchers identified an association of this polymorphism and lower risk of development CKD, acting as an effective protector factor of diabetic nephropathy in type 2 diabetes mellitus [23]. Other publications have demonstrated lower risk of CKD progression also [24], particularly in elderly [8]. Conversely, E4 allele have been associated with a more unfavorable metabolic profile in one study [25] and, according to *Song et al.*[26], it was associated with higher CAD risk. *Olivieiri* et al. [3] described E4 allele prevalence 2-fold higher on Mets (+) than MetS (−) population. Nevertheless, some studies did not showed the same association and the results were not representative of general population.

One possible explanation would be that the ApoE E4 allele polymorphism could be conditioned to other clinical variables presence, conferring, as along in our study, to MetS, an apolipoprotein modulator paper. This result suggests an association of this clinical condition in relation to ApoE polymorphism behavior.

This study has some limitations. It’s a cross sectional study, incapable for prospective analysis. Study groups sample size may confer lower reproducibility. The results of a study composed exclusively by hypertensive patients, of a unique center, cannot be extrapolated for the general population. Therapeutic effects and possible pharmacological interactions analysis along with the final results were not done, once medicine therapies were not interrupted or paused for ethical reasons. Genetic analysis methodology (SNP) has limitation on the genotype - phenotype interface comprehension: it represents part of the genetic predisposition; and, this methodology don’t enable the multiple numerous simultaneous genes analysis, like on genome wide association (GWA), hampering the search of a genetic signature for polygenetic diseases like MetS.

## Conclusions

In summary, the results presented in this study show that association between ApoE gene and CVD maybe modulated by the presence of MetS, with an increased CV burden observed among E4 carriers with the syndrome. On the opposite way, E4 carriers without visceral obesity had a lesser prevalence of CVD. The extrapolation of such observations shall be validated in a longitudinal observation and a larger population study.

## Methods

The study was approved by the Ethics and Research Committee (no. 311.413/06.21.2013, Ministry of Health, Brazil). Invited patients received explanations about study objectives and signed Ethics and Research Committee Informed Consent. It was enrolled 450 consecutive patients from the Hypertension and Cardiovascular Metabology Integrated Center of the Kidney and Hypertension Hospital, Universidade Federal de São Paulo (UNIFESP). Of the total, 67 patients were excluded because of not having completed sample collection and fulfilling Apo E polymorphism identification or because of participation refusal. This way, 383 patients were divided in two groups, classified by MetS presence, according to IDF definition: Group 1: 266 patients with MetS (MetS +) and Group 2: 117 patients without Mets (MetS -). Patient’s data were collected by clinical evaluation, physical exam, file reviews and laboratory testing. Presence of cardiovascular disease (CVD) was considered when identified on the office or on files registry, history of stroke, peripheral vascular disease, coronary artery disease or congestive heart failure [27]. Additionally, identification of diabetes mellitus and/or dislipidemia among patients were determined the *American Diabetes Association guideline*[28] and the *ACC/AHA 2013 Guideline for diagnosis and treatment of dyslipidemia*[29] definitions.

Glucose, total cholesterol and triglycerides plasmatic concentrations were determined by automatized enzimatic assay. HDL-cholesterol and LDL-cholesterol measures were determined by following commercial manufacturers specifications. Renal function was accessed by plasmatic urea level by colorimetric-enzimatic assay and by plasmatic creatinine level by Jaffé method. C-reactive protein was determined by quimioluminescence imune-assay.

### Analysis of apolipoprotein E gene polymorphisms

The sequence of 244 base pairs of the Apo E gene was amplified, through PCR amplification, using the primers:

*Sense*: 5′-TCCAAGGACCTGCAGGCGGCGCA-3′

*Antisense*; 5′ACAGAATTCCGCCCCGGCCTGGTACACTGCCA-3′

The PCR products were digested with the restriction enzyme Hha I and the fragments were separated by electrophoresis on 10% polyacrylamide gel. After running the gel was incubated with ethidium bromide for 30 minutes and the DNA fragments were visualized UV light. The E2, E3 and E4 alleles were analyzed and the corresponding genotypes.

Once obtained PCR product, 10ul were subjected to electrophoresis on 2% agarose gel stained with ethidium bromide (0.5 uL/mL) for 90 minutes at 120 V in 1× TBE solution. The specific amplification bands were visualized under ultraviolet (UV) light and photographed for later analysis of the polymorphism.

Groups were analyzed and compared on epidemiological, clinical and laboratorial characteristics as well as allele and genotype distribution in relation to ApoE polymorphism. The prevalence of different alleles and genotypes were analysed in relation to race observing the Hardy-Weinberg equilibrium. Groups were classified based on allele presence allowing analysis of clinical and laboratory characteristics. Prevalence of CVD, according to Mets presence, was analysed based on the three common alleles (E2,E3,E4) prevalence.

### Statistical analysis

Data were expressed in mean and standard deviation for variables with normal distribution. Student *t* test (parametric variables) and Mann–Whitney test (non parametric variables) were used to access, between groups, differences on analyzed variables. *X*^*2*^ test was used for proportional comparisons. Binary logistic regression was used to analyze the association between specific alleles manifestations of each polymorphism in relation to CVD presence. In all tests, 0,05 or 5% was fixed as the level of nulitiy hypotesis rejection.

## Abbreviations

Mets: Metabolic syndrome
ApoE: Apolipoprotein E
CVD: Cardiovascular disease
PCR: Polymerase chain reaction
WHO: World Health Organization
CRP: C-reactive protein
HDL: HDL-Cholesterol
Apo B: Apolipoprotein B
CAD: Coronary artery disease
BMI: Body mass index
ACEI: Angiotensin-Converting-Enzyme Inhibitor
ARB: Angiotensin II Receptor Blocker
DM: Diabetes Mellitus
LDL: LDL-cholesterol
CKD: Chronic kidney disease
SNP: Single nucleotide polymorphism
GWA: Genome Wide Association.
